# High expression of Stabilin-2 predicts poor prognosis in non-small-cell lung cancer

**DOI:** 10.1080/21655979.2021.1943109

**Published:** 2021-07-06

**Authors:** Juanjuan Yong, Liyun Huang, Gengbiao Chen, Xiaoya Luo, Hui Chen, Lin Wang

**Affiliations:** a Pathology Dept. Of Sun Yat-Sen Memorial Hospital of Sun Yat-Sen University, Guangzhou, Guangdong Province, China; b Pathology Dept. Of Sun Yat-sen University Cancer Center of Sun Yat-Sen University, Guangzhou, Guangdong Province, China; c Obstetrics and Gynecology Dept. Of Sun Yat-sen Memorial Hospital of Sun Yat-sen University, Guangzhou, Guangdong Province, China

**Keywords:** Stabilin-2, non-small-cell lung cancer, prognosis

## Abstract

Stabilin-2 has been found to regulate the progression of cancer. It was not fully understood whether it shows some roles in non-small-cell lung cancer (NSCLC). We used the immunohistochemical staining to evaluate Stabilin-2 protein expression in formalin-fixed parafﬁn-embedded tissue of NSCLC patients’ primary lesion. And we carried out χ2 test to detect relationships between Stabilin-2 expression and various clinical factors. Besides, the survival difference between patients with high and low Stabilin-2 expression was also analyzed. The expression of Stabilin-2 was associated with N stage and age. Higher Stabilin-2 expression exists in poorer survival patients. It revealed that Stabilin-2 expression was a significant predictor for both OS and DFS by univariate and multivariate analyses. High stabilin-2 expression in NSCLC predicts poor tumor prognosis.

## Graphical abstract

**Figure:**
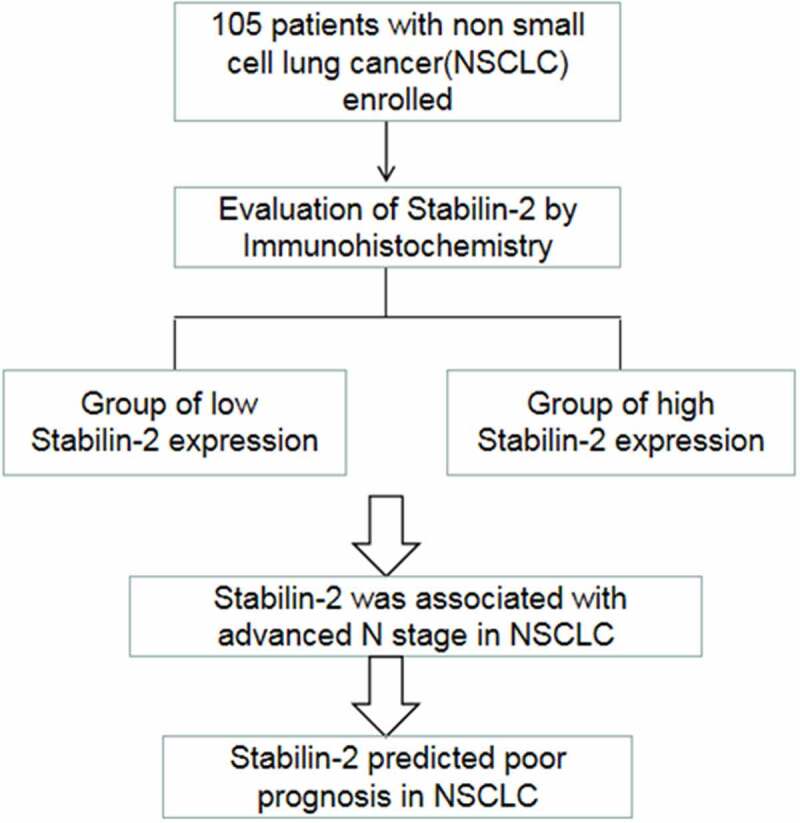

## Introduction

Lung cancer, as a common malignant tumor, has caused a great number of deaths every year [1,2]. Within lung cancer, the most prevalent subtype is non-small-cell lung cancer (NSCLC) [3,4]. Most patients suffer from an advanced lung cancer at their first visit because of the untypical clinical manifestations of NSCLC in its initial stage [5,6]. Although various treatment modalities such as surgery, radiotherapy, chemotherapy, and even target therapy showed undesired therapeutic effect. The prognosis of NSCLS was poor with 5-year OS less than 20% [7,8]. For the sake of improvement in NSCLC patients’ treatment efficacy, it is essential to investigate the molecular mechanisms underlying NSCLC development and progression. Currently, some molecular mechanisms such as EGFR have been found to be vitally important for the NSCLS and some therapy targeting these molecular mechanisms has been successfully used in the clinics [9–11]. However, more targets and new mechanisms are still in need to be found due to complexity of tumor biological behavior.

Stabilin-2 which is encoded by STAB2 gene acts as a clearance receptor for hyaluronic acid [12–14]. It has been reported that hyaluronic acid could affect the progression or metastasis of tumor cells. Thus, Stabilin-2 may also participate in regulating the cancer progression though affecting the level of hyaluronic acid [15]. STAB2 has been found to have the role to aid the diagnosis of malignant disease in prostate cancer patients. It is reported that expression of STAB2 in prostate cancer is correlated with patients’ advanced stage [16]. Besides, Stabilin-2 could also indicate the status lymph node metastasis in tongue cancer and other solid tumors such as lung, stomach and colorectal cancers [17]. What roles of Stabilin-2 on earth in NSCLC have not been well characterized.

We hypothesized that Stabilin-2 may lead to lymph node metastasis or poor tumor prognosis in NSCLC patients. In order to uncover its role as an oncogene in NSCLC, we performed our current study by analyzing a cohort of non-small-cell lung cancer (NSCLC) patients.

## Methods

### Ethics statement

This study was carried out according to the procedure made by the Ethics Committee of Sun Yat-sen Memorial hospital. Besides, the written informed consent was signed by each patient analyzed in our study.

### Patients and tumor tissue samples

We collected a total of 105 formalin-fixed parafﬁn-embedded tissue of NSCLC patients’ primary lesion from Sun Yat-sen Memorial Hospital and Sun Yat-sen University Cancer Center from the year 2012 to 2016. All the patients received no preoperative radiotherapy or chemotherapy. Surgery was performed with pathology confirmed of non-small-cell lung cancer. The clinical staging was evaluated according to the American Joint Committee on Cancer TNM staging system (8th Edition). After surgery, adjuvant treatment was scheduled according to the NCCN guidelines. Platinum containing dual drug regimen was used in the adjuvant chemotherapy. In patients with non-squamous cell pathologic subtype, pemetrexed disodium (500 mg/m^2^) plus cisplatin (75 mg/m^2^) or its analogues was used. While, for patients with squamous cell pathologic subtype, docetaxel (75 mg/m^2^) plus cisplatin (75 mg/m^2^) or its analogues was scheduled in these patients. All the patients who had indication to receive chemotherapy have completed four cycles of it. Formalin/PFA-fixed paraffin-embedded NSCLC resection tissue microarray blocks were made for Hematoxylin and eosin-stained slides and immunohistochemistry (IHC) staining.

### Immunohistochemistry

Manual and automatic immunohistochemical staining were both used in our study. Manual immunohistochemical staining methods: the tissue microarray slides should be baked in the thermostat for 40 min at a temperature of 60 degree. Then pathological tissue microarray was placed in xylene for 10 minutes and followed by another 10 minutes of xylene immersing. After deparaffinizing, the tissue microarray was rehydrated with graded ethanol (absolute ethanol, 95% ethanol, 75%ethanol and 50% ethanol, 5 minutes for each graded ethanol). The EDTA (1 mmol/L, pH 8.0) was used for antigen retrieval. We made these tissue microarray slides immersed in EDTA and then boiled for 10 minutes in a microwave oven. Next, 0.3% hydrogen peroxide we used to block the endogenous peroxidase was incubated for 15 minutes at room temperature about 25°C. After blocking with immunohistochemical blocking buffer for another 30 minutes, the tissue microarrays were incubated with the primary antibody (Rabbit anti-Stabilin-2 Polyclonal Antibody, ab121893, Abcam, Cambridge, UK, at a 1:300 dilution) overnight in a humidified chamber at 4°C. After washing and immersing for several minutes in PBS, the tissue microarrays were incubated with horseradish peroxidase-conjugated secondary antibody at 37°C for one hour. Finally, we used 3, 3ʹ-diaminobenzidine tetrahydrochloride (DAB) for signal production. Usually, it takes 3–5 minutes to develop the brown yellow color. Then, the tissue microarray slides were washed by PBS followed by nuclear staining with Hematoxylin for another 2 minutes.

Automatic immunohistochemical staining was done based on the tissue microarray sections in a Leica Bond MAX Immunostainer (Leica microsystem, Wetzlar, Germany). The primary antibody, a Rabbit Polyclonal Anti-Stabilin2 Antibody (ab121893, Abcam, Cambridge, UK) used, was diluted at 1:300. We used the Bond Refine-HRP detection system (DS9800, Leica microsystem, Wetzlar, Germany), including ER2 about 20 minutes. After the staining, we washed the sections with running water and distill water, then soaked in 95% alcohol for 1 min. Finally, they were dried in a 60°C oven about 60 min and sealed with neutral gum.

Negative and positive control tissues were on the same sections to make sure that the experiments were performed under the same experimental conditions for each sample.

### Semi-quantitative method

Since Stabilin-2 is usually expressed in the cell membrane, Cytoplasm. Only a small amount appears to be present at the cell surface. Any tumor cell membrane or cytoplasm staining was considered to be positive. Stabilin-2 protein levels by IHC detection were determined by the numbers of positive tumor cells and the staining intensity. The intensity and percentage of positively immunostaining tumor cells were separately assessed by two senior pathologists, using optical microscope and not affected by clinicopathologic data. If there were inconsistencies, the two senior pathologists reviewed them together and negotiated to produce relatively consistent results. The tumor cells percentage of positively cell membrane or cytoplasm staining was scored: ‘0’ meant no tumor cell staining, ‘1’ meant positively immunostaining tumor cells< 25%, ‘2’ meant positively immunostaining tumor cells 25%-50%, ‘3’ meant positively immunostaining tumor cells 50%-75% or focal tumor tissue stained, ‘4’ meant positively immunostaining tumor cells> 75%. The immunostaining intensity was scored: ‘0’ meant no cell membrane or cytoplasm staining, ‘1’ meant weakly cell membrane or cytoplasm stained, ‘2’ meant moderately cell membrane or cytoplasm stained, and ‘3’ meant strongly cell membrane or cytoplasm stained. The final Stabilin-2 immunostaining scores were defined as the percentage positive score plus the staining intensity score thus, the scores ranged from 1 to 7. On the basis of the expression level of Stabilin-2, patients involved were divided into two groups: the low Stabilin-2 expression group (score 1–4) and the high Stabilin-2 expression group (score 5–7).

### Follow up

When all treatments are finished, the follow up is initiated. In the first two years, patients were suggested to visit their physician every three months. The interval between two follow-up visits was extended to six months since the third year. During the five years when patients have been followed up, they just need to take the examination annually. During each follow up, laboratory examinations, chest CT scan and abdominal ultrasound were routinely performed. If patients showed some sign of recurrence, PET/CT and MRI was suggested to aid the diagnosis. In this study, Overall Survival (OS) meant the duration from the first surgical resection to death or last follow up; Disease-Free Survival (DFS) refers to the time interval from the first surgical resection to the tumor recurrence or last follow up.

### Statistical analysis

We used SPSS software for the statistical analysis of all valid data in this study. We used the χ2 test to find out the relationship between Stabilin-2 expression level in NSCLC patients’ primary lesion and various clinical factors. The survival curves drawing was done by using Kaplan-Meier Method. And the Log-Rank Test was applied for comparing the survival difference between study groups. Univariate and multivariate analyses which were performed through Cox proportional-hazard model was employed to acquire significant factors associated with survival. Statistically significant difference exists when P-value < 0.05.

## Results

In the present study, we hypothesized that Stabilin-2 may lead to lymph node metastatic invasiveness or poor prognosis in non-small-cell lung cancer (NSCLC) patients. In order to uncover its role as an oncogene in NSCLC, we detected the expression of Stabilin-2 in 105 NSCLC patients by using Immunohistochemistry. The detection results suggested that the expression level of Stabilin-2 was closely related to N stage in NSCLC. Patients in high Stabilin-2 expression group had a poorer survival time than those in low Stabilin-2 expression group. It was indicated that Stabilin-2 expression level was a significant predictor for both OS and DFS of patients suffered from NSCLC by using multivariate analysis.

### Baseline information of the patients enrolled

A total of 105 NSCLC patients were enrolled in, including 23 females and 82 males. The median age showed 60-year-old. Adenocarcinoma was the major pathologic subtype, which accounted for a percentage of 69.5%. Others were squamous carcinoma,bronchioloalveolar carcinoma, adenosquamous carcinoma. As for the tumor stage, T1-2 and T3-4 were found in 77 (73.3%) and 28 (26.7%), respectively. Lymph node metastasis were detected in 51 patients and the other 54 patients were lymph node negative. There were only 56 patients who underwent adjuvant chemotherapy while the other 49 patients did not (Table 1).

**Table 1.** Baseline characteristics of the patients

Variable	Number	Percent (%)
**Age, year**		
<60	54	51.4%
≥60	51	48.6%
**Pathologic subtype**		
Adenocarcinoma	73	69.5%
Other*	32	30.5%
**Gender**		
Male	82	78.1%
Female	23	21.9%
**Smoking**		
No	38	36.2%
Yes	67	63.8%
**T stage**		
1–2	77	73.3%
3–4	28	26.7%
**N stage**		
Negative	54	51.4%
Positive	51	48.6%
**TNM stage**		
I–II	60	57.1%
III–IV	45	42.9%
**Adjuvant chemotherapy**		
No	49	53.3%
Yes	56	46.7%

*:squamous carcinoma; bronchioloalveolar carcinoma; and adeno-squamous carcinoma.

### The relationship between Stabilin-2 expression and clinical factors in NSCLC

In our study, 58 cases showed low Stabilin-2 expression and 47 cases presented high Stabilin-2 expression. Our results demonstrated that expression of Stabilin-2 was correlated with N stage and age, but not with T stage, pathologic subtype and gender. In details, patients with N2-3 had a higher percentage of high Stabilin-2 expression than those with N1 stage. And the elderly patients were more likely to have high expression of Stabilin-2 than the young patients (Table 2).

**Table 2.** Relationship between Stabilin-2 expression and clinicopathological parameters

Variable	Low expression of Stabilin-2	High expression of Stabilin-2	*P* value
**Age, year**			0.042
<60	35	19	
≥60	23	28	
**Pathologic subtype**			0.773
Adenocarcinoma	41	32	
Other*	17	15	
**Gender**			0.539
Male	44	38	
Female	14	9	
**Smoking**			0.103
No	17	21	
Yes	41	26	
**T stage**			0.274
1–2	45	32	
3–4	13	15	
**N status**			0.042
Negative	35	19	
Positive	23	28	
**TNM stage**			0.126
I–II	37	23	
III–IV	21	24	
**Adjuvant chemotherapy**			0.979
Yes	31	25	
No	27	22	

*:squamous carcinoma; bronchioloalveolar carcinoma; and adeno-squamous carcinoma.

### High expression of Stabilin-2 was associated with impaired survival

**Figure 1.**
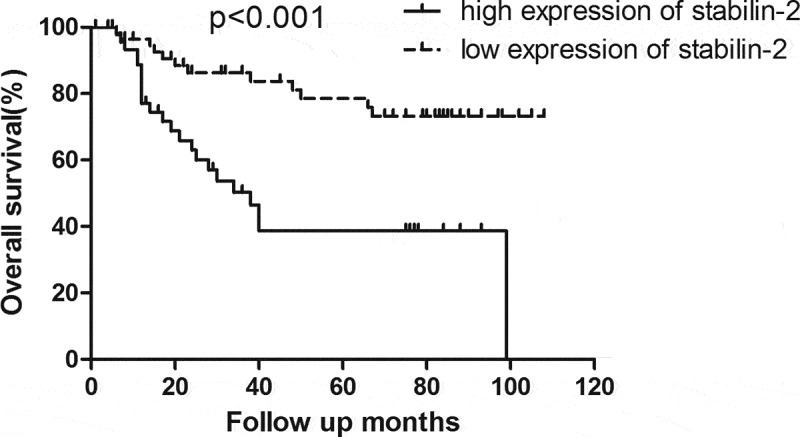
Patients with high expression of Stabilin-2 had a poorer OS than those with low expression of it (p < 0.001)

**Figure 2.**
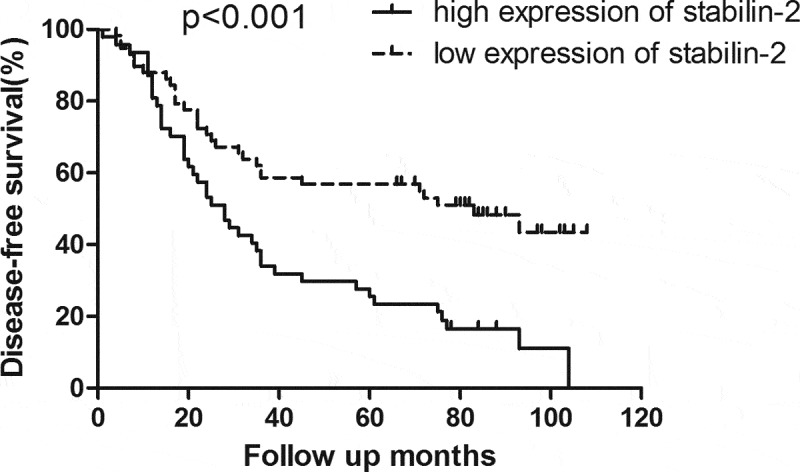
Patients with high expression of Stabilin-2 had a poorer DFS than those with low expression of it (p < 0.001)

The relationship between Stabilin-2 and tumor prognosis in NSCLC patients was analyzed by comparing the survival between these patients of low and high Stabilin-2 expression. Patients with low Stabilin-2 expression had better OS than did those with high Stabilin-2 expression (Figure 1). Besides, the DFS was also found to be higher in patients with low expression of Stabilin-2 than those with high expression of it (Figure 2).

### Univariate and multivariate analyses of prognostic factors in NSCLC patients

By performing univariate analyses, we showed that stabilin-2 and N stage were significantly associated with OS and DFS in these **NSCLC** patients, while, other variables, including age, gender, adjuvant chemotherapy and T stage, were not found to be correlated with OS and DFS (Table 3). Besides, multivariate analyses were further done and the results also revealed that stabilin-2 and N stage were significant predictors for both OS and DFS. Thereby, Stabilin-2 expression may be useful in predicting the tumor prognosis in patients with NSCLC (Table 4).

**Table 3.** Univariate analysis of OS and DFS for the whole group

variable	Number of patients	3-year OS	*P* value	3-year DFS	*P* value
**Age, year**			0.535		0.445
<60	54	70.4%		51.9%	
≥60	51	70.5%		43.1%	
**Pathologic subtype**			0.531		0.533
Adenocarcinoma	73	70.9%		47.9%	
Other*	32	70.0%		46.9%	
**Gender**			0.807		0.896
Male	82	70.9%		47.6%	
Female	23	70.6%		47.8%	
**T stage**			0.664		0.535
1–2	77	70.0%		49.4%	
3–4	28	71.6%		42.9%	
**N status**			0.002		<0.001
Negative	54	83.9%		64.8%	
Positive	51	54.2%		29.4%	
**Adjuvant chemotherapy**			0.134		0.01
Yes	56	74.1%		58.9%	
No	49	66.4%		34.7%	
**Expression of Stabilin-2**			<0.001		<0.001
Low	58	86.3%		58.6%	
High	47	50.3%		34.1%	

Abbreviation: DFS, disease-free survival; OS, overall survival.

**Table 4.** Multivariate analysis of DFS and OS for all the patients

Variable	OS		DFS	
	HR (95%CI)	*P* value	HR (95%CI)	*P* value
**Expression of Stabilin-2 (low vs high)**	0.333 (0.162–0.683	0.003	0.507 (0.311–0.824)	0.006
**N status (negative vs positive)**	0.434 (0.213–0.884)	0.022	0.457 (0.278–0.751)	0.002

Abbreviations: DFS, disease-free survival; OS, overall survival; CI, confidence interval; HR, hazard ratio.

## Discussion

In our current study, we have uncovered the role of stabilin-2 in NSCLC. First, we detected the expression of Stabilin-2 by performing immunohistochemistry in a cohort of NSCLC patients. We showed that higher expression of Stabilin-2 was correlated with higher N stage. And this result just suggested that Stabilin-2 might act as an oncogene in stimulating the progression of non-small-cell lung cancer. Next, we performed survival analysis by using Kaplan-Meier Method and we found that the expression of Stabilin-2 was significantly associated with DFS and OS in NSCLC patients. The 3-year DFS for groups with high and low expression of Stabilin-2 were 34.1% and 58.6%, respectively. Obvious difference existed between the two studied groups. Besides, compared to patients with low expression of Stabilin-2, those with high expression of it had an impaired overall survival as well.

Further multivariable analysis was performed to confirm the results acquired from univariable analysis. Our data demonstrated that Stabilin-2 expression, together with N stage, was an independent predictor of DFS and OS of these patients. All the results just suggested that Stabilin-2 expression could help define a subset of patients with unfavorable prognosis and might be a potential treatment target for lung cancer patients.

In solid tumors such as tongue, lung, and stomach cancers, Stabilin-2 tended to be expressed in the sinusoidal endothelial cell within the metastatic lymph nodes [18]. Especially in tongue cancer, Stabilin-2 was found to be correlated with lymph node metastasis and the tumor recurrence. In hepatocellular carcinoma, endothelial transdifferentiation is a major pathogenic event and stabilin-2 could accelerate endothelial-tumor cell adhesive interactions and microvascular invasion. Thereby, loss of Stabilin-2 expression showed increased survival in patients with liver cancer [18]. Stabilin-2 has been considered as a major clearance receptor for hyaluronic acid (HA) which shows a role in inhibiting the metastasis of tumor cells. Thus, blocking the function of Stabilin-2 may inhibit the metastasis of tumor by improving the level of circulating HA [15]. Stabilin-2 has also been found to participate in regulating the retrial-venous differentiation. When Stabilin-2 was knocked down, the arterial-venous differentiation and axial vessel formation was impaired. And the decreased expression of Stabilin-2 also led to abnormal expression of venous markers [19]. The underlying mechanism was that Stabilin-2 could activate pERK1/2 pathway which was a transducer of VEGF signaling during the arterial-venous differentiation [20,21]. it was reported that, when HA was added to cells which express Stabilin-2, the phosphorylation of ERK1/2 also promoted [22].

Our study which was consistent with others also showed that high expression of Stabilin-2 was associated with advanced N stage [17]. Besides, we also found that the expression of Stabilin-2 was significant predictor of DFS and OS.

Some other metabolic and genetic factors have also been reported to show some roles in the carcinogenesis of NSCLC. For instance, decreased expression of p16 was frequently to be observed in adenocarcinoma of lung cancer. p16 functions as a tumor suppressor which can inhibit the phosphorylation of the retinoblastoma protein (pRb) and block the cell cycle [23,24]. In oropharyngeal cancer, high expression of p16 was correlated with a decreased EGFR expression, suggesting a relationship between p16 and EGFR activation [25]. Another important molecule was GLUT1. Its expression was found to be associated with Ki-67 expression and KRAS mutation in resect lung cancer [26,27]. GLUT1 stimulated the tumor growth by accelerating the metabolism of cancer cell in the anaerobic conditions. Usually, under the hypoxia circumstance, the fast metabolism of lung cancer cell needs more glucose uptake, which stimulate the VEGF-induced neoangiogenesis and activates SLC2A1 and A3 transcription which encodes GLUT1and GLUT3 [23]. From this point, Stabilin-2 may cooperate with GLUT1 in stimulating the progression of cancer cell under hypoxia condition since Stabilin-2 also showed a role in activating VEGF pathway.

There were still some flaws in this study. Firstly, the present study was a retrospective evaluation of Stabilin-2 expression in 105 patients and we could not eliminate the selection bias during the collection of patients’ samples. Secondly, our study lacked the evidence from vitro experiments and the underlying mechanism of Stabilin-2 in regulating tumor progression in NSCLC remained poorly understood.

## Conclusion

In conclusion, high expression of Stabilin-2 has significant correlations with lymph node metastasis and impaired survival in non-small cell lung cancer (NSCLC). However, further investigation on the mechanism of Stabilin-2 regulating the progression of non-small cell lung cancer (NSCLC) is still needed.

## Highlights

Stabilin-2 was correlated with advanced N stage in lung cancer patients;

High expression of Stabilin-2 predicted poor prognosis in non-small-cell lung cancer patients;

Stabilin-2 can be a potential therapeutic target for non-small-cell lung cancer.
